# Paeonol attenuates heart failure induced by transverse aortic constriction via ERK1/2 signalling

**DOI:** 10.1080/13880209.2022.2040543

**Published:** 2022-03-07

**Authors:** Xu Chen, Zhiyu Zhang, Xiaowei Zhang, Zhi Jia, Jun Liu, Xinpei Chen, Aiqing Xu, Xue Liang, Guangping Li

**Affiliations:** a Tianjin Key Laboratory of Ionic-Molecular Function of Cardiovascular Disease, Department of Cardiology, Tianjin Institute of Cardiology, The Second Hospital of Tianjin Medical University, Tianjin, China; b Department of Cardiology, Tianjin Beichen Hospital, Tianjin, China; c Tianjin Beichen Center for Disease Control and Prevention, Tianjin, China

**Keywords:** Paeonol, cardiac fibrosis, apoptosis, ERK1/2

## Abstract

**Context:**

Paeonol (PAE) is the main phytochemical from *Cortex Moutan*. Its main pharmacological effects are anti-inflammatory and antioxidant, but its cardioprotective effect is unclear.

**Objective:**

The study investigates the effects and underlying mechanisms of PAE on transverse aortic constriction (TAC)-induced heart failure (HF) in mice.

**Materials and methods:**

C57BL/6 mice were randomly divided into five groups: sham, TAC, PAE10 (TAC + PAE 10 mg/kg), PAE20 (TAC + PAE 20 mg/kg) and PAE 50 (TAC + PAE 50 mg/kg). Paeonol was intragastrically administered to mice for 4 weeks. Mice were anaesthetized with pentobarbital sodium and underwent cardiac echocardiography using echocardiography system. Serum levels of atrial natriuretic peptide (ANP), tumour necrosis factor-α (TNF-α) and interleukin-6 (IL-6) were measured by enzyme-linked immunosorbent assay (ELISA). Myocardial apoptosis was detected with terminal deoxynucleotidyl transferase-mediated dUTP nick end-labelling (TUNEL) staining. Haematoxylin–eosin (H&E) and Masson’s staining were used for histopathological evaluation. Western and quantitative real-time PCR (qRT-PCR) were performed to detect levels of apoptosis and fibrosis-related proteins.

**Results:**

Echocardiography showed PAE improved cardiac function (LVEF: TAC, 52.3±6.8%; PAE20, 65.8±3.6%; PAE50, 71.4±2.5%) and H&E staining showed PAE alleviated myocardial injury (TAC: 1170.3 ± 134.6 μm^2^; PAE50: 576.0 ± 53.5 μm^2^). Western and qRT-PCR results showed that PAE down-regulated the levels of ANP, BNP and α-MHC. In addition, TUNEL and western results showed PAE significantly inhibited apoptosis. Masson and western results showed PAE inhibited cardiac hypertrophy. Western results showed the ERK1/2/JNK pathway could be inhibited by PAE.

**Discussion and conclusions:**

Paeonol regulates ERK1/2/JNK to improve cardiac function, which provides theoretical support for the extensive clinical treatment of HF.

## Introduction

Heart failure (HF) is a multifactorial, degenerative and intractable disease, which is a leading cause of mortality and morbidity worldwide. Heart failure, accompanied by ventricular hypertrophy (Okin et al. 2004), cardiac apoptosis (Asselin et al. 2020), cardiac fibrosis and myocardial inflammation, is thought to be a common final stage in numerous cardiovascular diseases such as hypertension, myocardial infarction and myocarditis. Although surgical treatment and drug intervention can significantly reduce the mortality of patients, the incidence of the disease is still increasing year by year. Hence, it is critical to elucidate the molecular mechanism of HF and explore the new treatment strategies to prevent and treat HF.

Previous studies have demonstrated that members of mitogen-activated protein kinase (MAPK) family play a vital role in intracellular and extracellular signal transduction in different cells and regulate the important biological process, including differentiation, proliferation, migration and apoptosis (Sun et al. 2015). The progression of apoptosis under pathological conditions of myocardial ischaemia was accompanied by the activation of ERK1/2 signalling pathway (Yan et al. 2018). C-Jun N-terminal kinase (JNK) is thought to be involved in the induction of apoptosis by activating the cellular damage signalling pathway. JNK signalling pathway can be activated by inflammation, contributing to the terminal point of HF from transverse aortic constriction (TAC) to cardiac remodelling. Some studies have confirmed that the regulation of the JNK/Bcl-2 pathway may be the key mechanism of anthocyanin in alleviating myocardial infarction (Syeda et al. 2019). Based on a growing body of research, we hypothesized that the ERK1/2/JNK signalling pathway is involved in the development of TAC-induced HF.

Medicinal plants and natural compounds have traditionally been used to treat a variety of cardiovascular diseases (Jia et al. 2020). Paeonol (2′-hydroxy-4′-methoxyacetophenone, PAE) is an active ingredient extracted from the famous traditional Chinese herbal medicine *Cortex Moutan*. It is well known that PAE has extensive pharmacological activities, including antioxidant effects (Jin et al. 2016), anti-inflammatory, antiplatelet (Lu et al. 2018), anti-atherosclerotic and apoptosis inhibition (Gao et al. 2019). Studies have confirmed that PAE regulates the ERK1/2 signalling pathway and regulates the proliferation of PASMCs to play a role in the treatment of pulmonary hypertension (Zhang et al. 2018). Wang et al. (2011) found that PAE plays a neuroprotective role on glutamate neurotoxicity by inhibiting apoptotic signalling pathway and activating ERK pathway. Paeonol could suppress the myocardial apoptosis via activation of Nrf2/HO-1 signalling, and alleviate myocardial infarction-induced cardiac injury (Li et al. 2016). However, the potential effect of PAE on TAC-induced HF and the underlying signalling mechanism have not yet been revealed. In the present study, we found that PAE may alleviate HF through the regulation of the ERK1/2 signalling pathway.

## Materials and methods

### Reagents

Paeonol was purchased from Sigma-Aldrich (St. Louis, MO). Antibodies against to ANP, BNP, α-MHC, collagen I (Col I), connective tissue growth factor (CTGF), fibronectin-1, AKT, p-AKT, ERK1/2, p-ERK1/2, JNK, p-JNK and β-actin were purchased from Cell Signaling Technology (Boston, MA). Enzyme-linked immunosorbent assay (ELISA) kits for IL-6, TNF-α and ANP were purchased from R&D Systems Inc. (Minneapolis, MN).

### Animals

Adult male C57BL/6 mice were obtained from Beijing Huafukang Biotechnology Co., Ltd. (Beijing, China). The mice were housed in cages under controlled conditions, with a 12 h light/dark cycle, and a temperature of 22 ± 2 °C. The mice had free access to food and water. The mice were randomly divided into five groups (*n* = 10): sham, TAC, PAE10 (TAC + PAE 10 mg/kg), PAE20 (TAC + PAE 20 mg/kg) and PAE50 (TAC + PAE 50 mg/kg). The PAE dosage was determined according to the dosage used in a previous study (Wu et al. 2016). After surgery, mice were given gastric administration for 4 weeks. The laboratory technician made every effort to minimize the number of mice used and reduce the suffering. All experimental procedures were approved by the animal ethics committee of the Second Hospital of Tianjin Medical University on 1 December 2018.

### TAC model and treatment

The mice were anaesthetized with pentobarbital sodium (6 mg/kg, i.p.). TAC model was performed as previously described (Yasuno et al. 2013). Briefly, the chest cavity was opened to expose the aortic arch. Then, a 7-0 thread is used to attach the arch of the aorta between the artery and the left carotid to a 27-gauge needle. The needle was gently removed, causing approximately 70% contraction. Sham group mice underwent a similar surgery, except for the contraction of the aortic arch.

### Cardiac echocardiography

Four weeks after drug administration, mice were anaesthetized with pentobarbital sodium (6 mg/kg, i.p.) and underwent cardiac echocardiography using echocardiography system. The left ventricular ejection fraction (EF) and fractional shortening (FS) were calculated from the M-mode recording. The mean values of six different cardiac cycles were taken for all tests.

### Heart weighing

After 4 weeks of treatment, the mice were anesthetized using pentobarbital sodium, weighed, and their heart tissues were taken according to the unified standard, and the heart was weighed. Then, the ratio of heart weight/body weight (HW/BW) and the ratio of heart weight/tibia length (HW/TL) were calculated.

### Enzyme-linked immunosorbent assay kit

Blood samples were taken after anaesthesia. Serum levels of atrial natriuretic peptide (ANP), tumour necrosis factor-α (TNF-α) and interleukin-6 (IL-6) were measured by the ELISA kits (R&D Systems Inc., Minneapolis, MN), according to manufacturer’s instruction.

### Histopathology

Myocardial tissue was fixed in 4% formalin. The fixed tissues were embedded in paraffin, sectioned at 5 μm. Then, the sections were stained with haematoxylin and eosin (H&E) and Masson’s trichrome as described previously (Yang et al. 2005; Zhao et al. 2015). The images were examined under a light microscope.

### Western blotting assay

After anaesthesia, the heart was irrigated with PBS. Heart tissues were lysed in RIPA buffer. Proteins were isolated as described previously. BCA kit was used to detect the protein concentration. After dilution to the same concentration, the expression level of specific protein was detected by electrophoresis. The membranes were incubated with primary antibodies against ANP, BNP, α-MHC, AKT, p-AKT, ERK1/2, p-ERK1/2, JNK, p-JNK and β-actin (internal control) at 4 °C overnight. Blots were sequentially incubated with HRP-conjugated secondary antibodies at 25 °C. The protein bands were visualized using the ECL reagent. The densitometry was analysed using Image J software (Bethesda, MD).

### Quantitative real-time PCR (qRT-PCR) assay

qRT-PCR analysis was used to detect the mRNA expression of ANP, BNP, α-MHC, Col I, CTGF, fibronectin-1 and β-actin in heart tissue. RNA was isolated using TRIzol reagent as described previously (Zhang et al. 2013). The primer sequences were as follows: ANP, forward: 5′-GGCTTCTTCCTCGTCTTGG-3′, reverse: 5′-ATCTGTGTTGGACACCGCA-3′, BNP, forward: 5′-CGGTCTCAAGGCAGCAC-3′ reverse: 5′-GTTACAGCCCAAACGACT-3′, Col I, forward: 5′-CAATGGCACGGCTGTGTGCG-3′, reverse: 5′-CACTCGCCCTCCCGTCTTTGG-3′, CTGF, forward: 5′-CTTCTGCAGACTGGAGAAGC-3′, reverse: 5′-CAGCCAGAAAGCTCAAACTTG-3′, β-actin, forward: 5′-CCAACCGCGAGAAGATGA-3′, reverse: 5′-CCAGAGGCGTACAGGGATAG-3′. The mRNA levels were normalized to β-actin mRNA. The relative expression levels were calculated with the 2^–ΔΔCT^ method and three independent experiments were performed.

### Terminal deoxynucleotidyl transferase-mediated dUTP nick end-labelling (TUNEL) assay

After mice were sacrificed, the heart samples were fixed in 4% formalin for 24 h, subsequently, embedded in paraffin, sliced (5 μm). Next, the sections and the apoptotic myocardial cells were detected with a TUNEL assay kit according to the manufacturer’s instructions. TUNEL staining images in different fields were collected by an optical microscope. The TUNEL positive cells were calculated via Image J software (Bethesda, MD).

### Data analysis

SPSS 22.0 software (SPSS Inc., Chicago, IL) was used for statistical analysis. Data were presented as means ± standard deviation (means ± SD). One-way ANOVA followed by a *post hoc* test (least significant difference) was used to compare the differences among the different groups. Student's *t*-test was used to compare the differences between the two groups. *p* < 0.05 was considered statistically significant.

## Results

### Paeonol improved cardiac function and reduced ventricular hypertrophy in TAC mice

**Figure 1.**
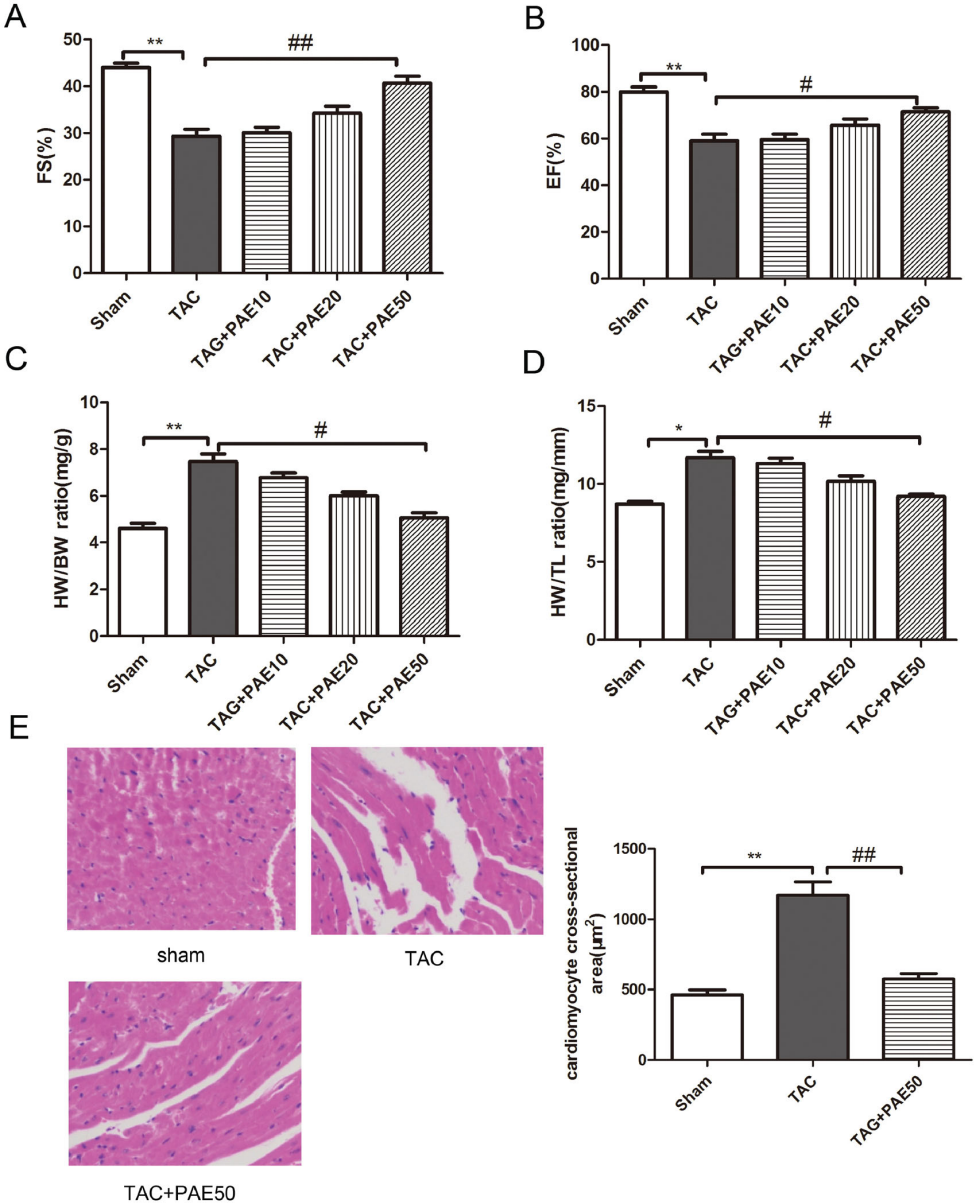
PAE improved cardiac function (EF, FS) and reduced ventricular hypertrophy (HE) in TAC mice. Echocardiography was used to evaluate cardiac function in different groups. PAE prevented the development of cardiac failure in TAC mice. Effect of PAE on FS (A), EF (B), heart weight/body weight (C) and heart weight/tibia length (D). Representative photographs of sections of left ventricle with H&E staining in the different groups (E), quantification of myocardial injury by H&E staining sections. Data were presented as mean ± SD vs. sham group, *p < 0.05, **p < 0.01; vs. TAC group, ^#^*p* < 0.05, ^##^*p* < 0.01, *n* = 6.

M-mode echocardiography was used to detect the cardiac function in the different groups after TAC surgery and PAE administration. As shown in Figure 1(A,B), the results showed that compared with sham group, FS and EF values were significantly reduced, hence which suggesting that HF in TAC mice. After 4 weeks treatment with PAE, FS and EF values were markedly recovered. The results verified that PAE could significantly improve cardiac function. Then we measured the heart weight, body weight and tibia length of mice in different groups. Results (Figure 1(C,D)) showed that compared with the sham group, the ratio of HW/BW and HW/TL in TAC group was increased obviously. While after the treatment, the ratio was reduced markedly in a dose-dependent manner. In addition, H&E staining was performed to assess the morphological changes of myocardial injury in different groups. As shown in Figure 1(E), it demonstrated that cardiomyocytes were normal and present in an orderly arrangement in sham group whereas, after TAC surgery, there was cardiac hypertrophy, disorder, as well as loss of normal structure. After treatment with PAE, compared with the TAC group, the cardiomyocytes were arranged more neatly and orderly, and there was no obvious damage. Analysis of myocardial injury confirmed that PAE suppressed TAC-induced cardiac hypertrophy. Taken together, it revealed that PAE could attenuate TAC-induced cardiac hypertrophy and myocardial injury.

### Paeonol reduced the expression of markers related to cardiac hypertrophy in TAC mice

**Figure 2.**
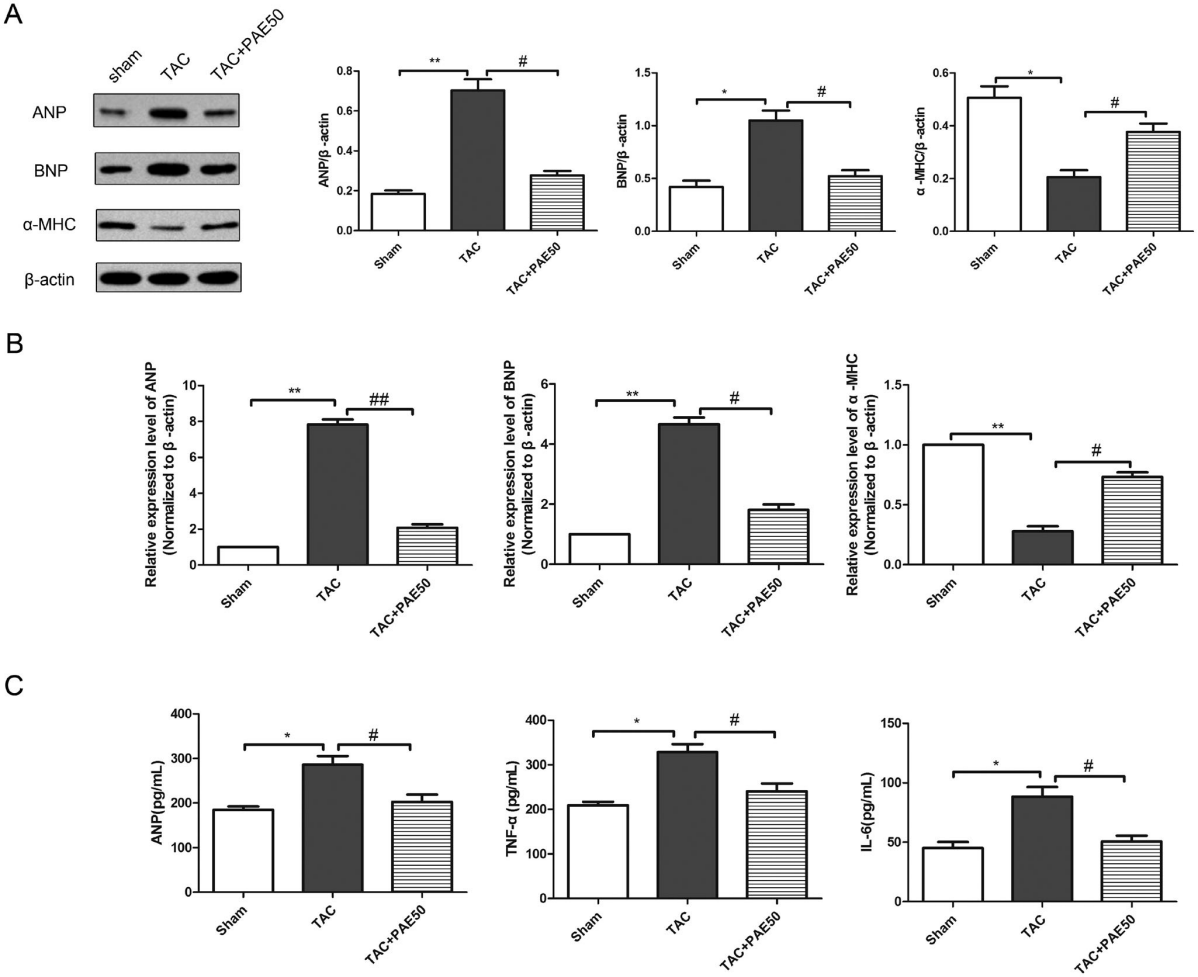
Paeonol reduced the expression of markers related to cardiac hypertrophy in TAC mice. Western blotting assay was performed to assess the expression of ANP, BNP and α-MHC in different groups. Representative blots normalized to β-actin expression were presented (A). The mRNA expression of ANP, BNP and α-MHC in different groups was measured by qRT-PCR analysis (B). The concentration of ANP, TNF-α and IL-6 in the serum was detected via ELISA kits (C). Data were presented as mean ± SD vs. sham group, *p < 0.05, **p < 0.01; vs. TAC group, ^#^*p* < 0.05, ^##^*p* < 0.01, *n* = 6.

It is well known that the level of ANP indicates changes in cardiac hypertrophy, and the levels of TNF-α, IL-6 and fibrin indicate myocardial inflammation and fibrosis induced by cardiac hypertrophy. To evaluate the effect of PAE on biochemical markers of TAC-induced HF, western blotting assay and qRT-PCR analysis were performed to detect the expression of ANP, BNP and α-MHC in the heart tissue of each group. As shown in Figure 2(A), the results showed that compared with sham group, the expression of ANP and BNP in TAC group was increased significantly, while the expression of α-MHC was reduced markedly. After treatment, PAE reversed the changes. Similarly, qRT-PCR results (Figure 2(B)) showed the same trend. Furthermore, we detected the relevant biochemical indicators in serum using ELISA kits. As shown in Figure 2(C), it showed that the concentration of ANP, TNF-α and IL-6 was significantly increased in TAC group. After treatment, PAE could markedly decrease the levels of ANP, TNF-α and IL-6. This confirmed that PAE reduced the expression of cardiac hypertrophy and serum biochemical indicators in TAC mice.

### Paeonol reduced fibrosis in TAC mice

**Figure 3.**
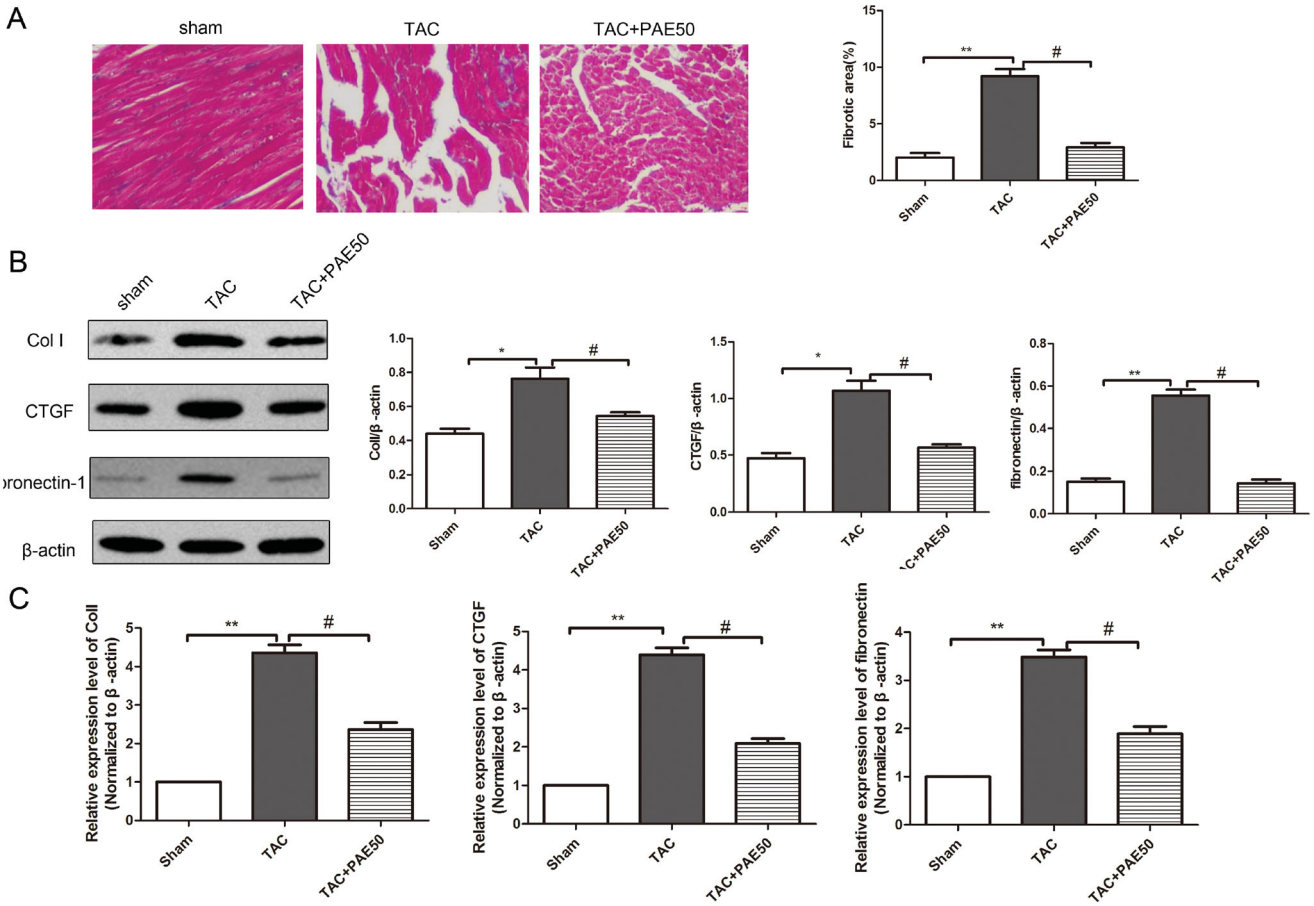
Paeonol inhibited fibrosis and reduced the expression of Col I, CTGF and fibronectin-1 in TAC mice. Representative photographs of sections of left ventricle with Masson trichrome staining in the different groups. Quantitative analysis of cardiac fibrotic area by Masson’s trichromatic staining section (A). The protein level of Col I, CTGF and fibronectin-1 in heart tissue was detected by western blotting assay. (B) Representative blots normalized to β-actin expression were presented. (C) The mRNA expression of Col I, CTGF and fibronectin-1 in heart tissue was detected via qRT-PCR assay. Data were presented as mean ± SD vs. sham group, *p < 0.05, **p < 0.01; vs. TAC group, ^#^*p* < 0.05, *n* = 6.

The pathological process of HF is accompanied by continuous myocardial fibrosis. Hence, we examined the inhibitory effect of PAE on TAC-induced HF fibrosis. Masson trichrome staining was used to detect the collagen deposits in TAC-induced mice. As shown in Figure 3(A), Masson’s staining results revealed that compared with sham group, the collagen deposits in TAC group were obviously increased. Fibrosis was inhibited by treatment with PAE. Next, western blotting assay and qRT-PCR assay were performed to detect the expression levels of fibrosis-related proteins and mRNA. The western blot results (Figure 3(B)) showed that the expressions of Col I, CTGF and fibronectin-1 in TAC group were increased significantly compared with the sham group. After treatment, the expressions of Col I, CTGF and fibronectin-1 were reduced markedly. Similarly, qRT-PCR analysis results (Figure 3(C)) showed the same trend. Taken together, it revealed that PAE inhibited fibrosis in TAC mice.

### Paeonol reduced cardiac apoptosis in TAC mice

**Figure 4.**
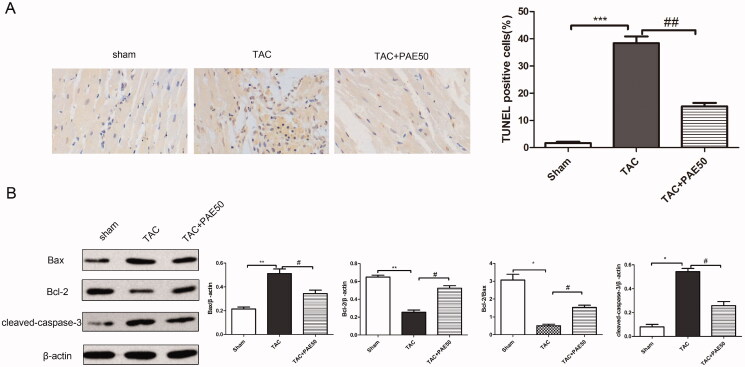
Paeonol reduced cardiac apoptosis in TAC mice. (A) Representative photographs of TUNEL staining in heart sections. Quantitative analysis of TUNEL staining in heart sections. (B) Western blotting assay was performed to assess the expression of Bax, Bcl-2 and cleaved-caspase-3 in heart tissue. Representative blots normalized to β-actin expression were presented. Data were presented as mean ± SD vs. sham group, *p < 0.05, **p < 0.01, ***p < 0.001; vs. TAC group, ^#^*p* < 0.05, ^##^*p* < 0.01, *n* = 3.

To evaluate the effect of PAE on cardiac apoptosis in TAC mice, TUNEL staining was performed to assess the apoptosis in different groups. Western blotting assay was used to detect the expression of apoptosis-related proteins in heart tissue. As shown in Figure 4(A), TUNEL staining results showed that the TUNEL positive cells in TAC group significantly increased compared with sham group, which indicated the significant apoptosis in TAC group. After treatment with PAE, TUNEL positive cells markedly decreased. Additionally, western blot results (Figure 4(B)) showed that the ratio of Bcl-2/Bax and the expression of cleaved-caspase-3 were increased significantly in TAC group compared with sham group. On the contrary, the ratio of Bcl-2/Bax and the expression of leaved-caspase-3 in the treatment group were reduced remarkably. Taken together, it demonstrated that PAE alleviated cardiac apoptosis in TAC mice.

### Paeonol inhibited ERK1/2/JNK signalling pathway in TAC mice

**Figure 5.**
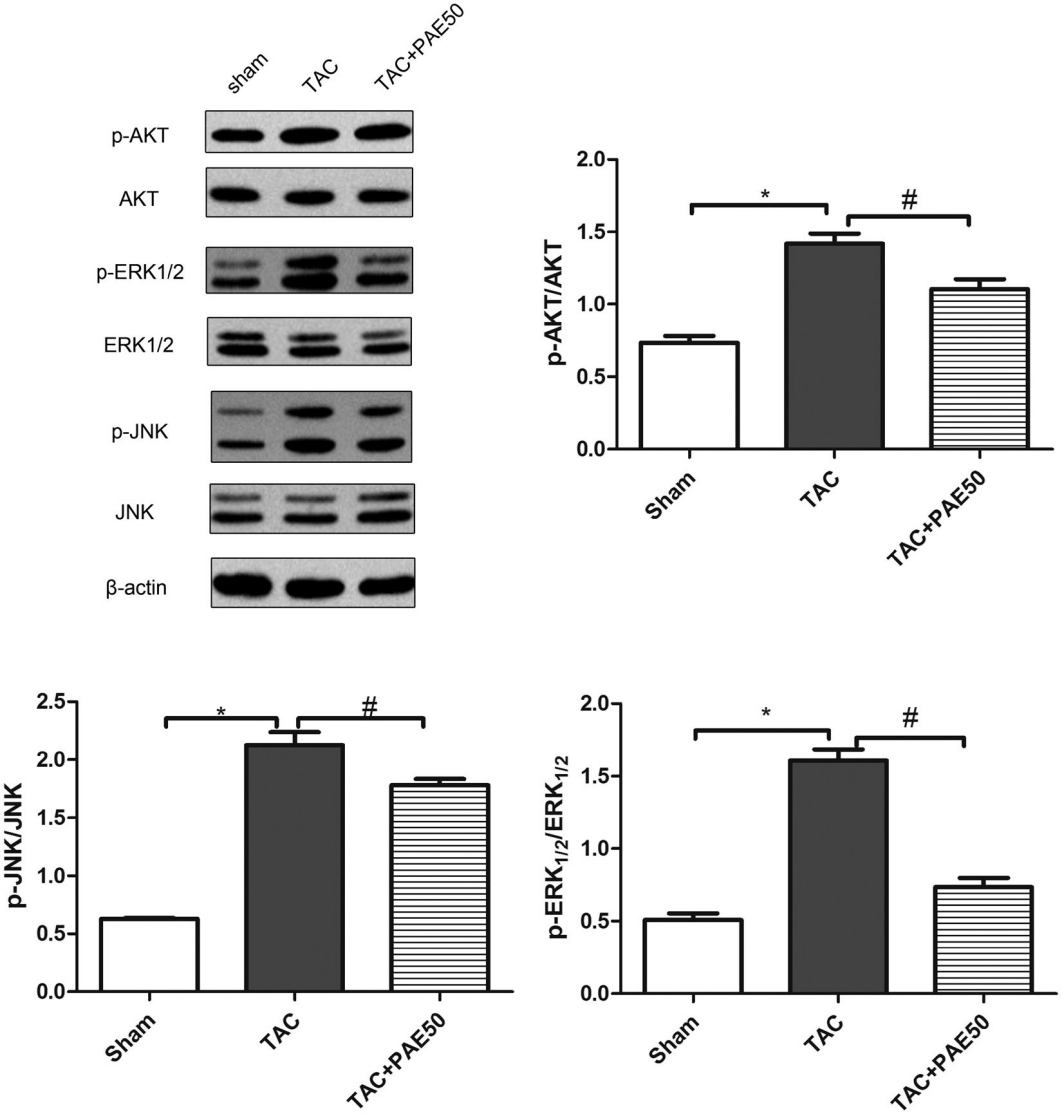
Paeonol inhibited the expression of p-ERK1/2, p-AKT and p-JNK in TAC mice. Western blotting assay was used to detect the ratio of p-AKT/AKT, p-ERK1/2/ERK1/2 and p-JNK/JNK in heart tissue. Representative blots normalized to β-actin expression were presented. Data were presented as mean ± SD vs. sham group, *p < 0.05; vs. TAC group, ^#^*p* < 0.05, *n* = 3.

Western blotting assay was used to detect the expression of AKT, p-AKT, ERK1/2, p-ERK1/2, JNK and p-JNK in heart tissue. As shown in Figure 5, western blot results showed that the ratio of p-AKT/AKT, p-ERK1/2/ERK1/2 and p-JNK/JNK in TAC group was increased significantly compared with sham group. After treatment, it down-regulated the expression of p-AKT, p-ERK1/2 and p-JNK. The results suggested that PAE inhibited ERK1/2/JNK signalling pathway in TAC mice.

### Paeonol attenuates activation of ERK1/2 signalling in TAC mice

To explore the pathway through which PAE exerts its anti-fibrotic effects, we assessed the changes in expression of the fibrotic mediators using Western blot analysis. Since ERK1/2/JNK are the most well-known pro-fibrogenic mediators, we first examined the effects of PAE on p-ERK1/2 and p-JNK protein levels. As shown in Figure 5, compared with the sham group, the expression of p-AKT, p-ERK1/2 and p-JNK in the TAC group was increased significantly, while after treatment, the expression decreased markedly. Studies have shown that PAE attenuated TAC-induced myocardial injury through the activation of ERK1/2 signalling in TAC mice.

## Discussion

A variety of cardiovascular diseases with HF as the critical endpoint are posing a serious threat to human health and greatly increasing the medical and economic burden worldwide. For thousands of years, the effective clinical application of traditional Chinese medicine in the treatment of complex cardiovascular diseases and other difficult diseases has attracted the attention of a large number of researchers (Yang et al. 2019). It has been reported that PAE, a traditional Chinese medicine, has antioxidant, apoptosis inhibition, resistance to myocardial ischaemia injury (Li et al. 2016). In the present study, we have observed the protective effect and potential signalling mechanism of PAE in treatment of TAC-induced HF. The results showed that PAE could significantly reduce cardiac hypertrophy, inhibit cardiac apoptosis, thus alleviating the progression of TAC-induced HF.

First, we demonstrated that high doses of PAE (50 mg/kg) significantly reduced the ratio of HW/BW and HW/TL, and the values of FS and EF were markedly recovered in TAC-induced mice. Up-regulation of ANP, BNP and α-MHC has been reported in cardiac hypertrophy and HF (Xu et al. 2020). In other words, the expression of ANP, BNP and α-MHC was significantly increased in serum of TAC-induced mice, and they was significantly down-regulated after treatment with PAE in the study. Consistent with the previous study (Choy et al. 2018), combined with H&E staining results, we suggested that PAE could alleviate myocardial hypertrophy and improve cardiac function.

Results of ELISA kits showed that PAE could reduce the levels of TNF-α and IL-6 in serum of TAC mice, suggesting that PAE could inhibit the inflammatory response in TAC mice. In agreement with the previous report, PAE can attenuate kidney injury in septic mice by inhibiting HMGB1 mediated inflammatory response (Mei et al. 2019). Wu et al. (2018) found that PAE improved cardiac dysfunction, alleviated inflammation and reduced myocardial apoptosis. In our study, TUNEL results confirmed that increased cardiac apoptosis in TAC mice and marked downregulation of apoptosis after treatment. It suggested that inhibition of inflammatory response and apoptosis may be the mechanisms of PAE alleviating TAC-induced HF.

Furthermore, similar to cardiomyocyte hypertrophy, cardiac fibrosis is a key pathological process in the progression of HF (Bacmeister et al. 2019). Increased collagen deposition in and around the blood vessels and myocardium can impair cardiac function (Suematsu et al. 2018). Therefore, inhibition of cardiac fibrosis is an important target in the treatment of HF. In the present study, Masson’s staining results showed that PAE significantly reduced collagen deposition after treatment, indicating that PAE can inhibit cardiac fibrosis. The expressions of Col I, CTGF and fibronectin-1 in TAC mice were significantly increased and were significantly down-regulated after treatment. Taken together, the data confirmed that PAE could inhibit myocardial fibrosis and alleviate the pathological process of TAC-induced HF. Zhao et al. (2020) found SMYAD inhibited cardiac hypertrophy and fibrosis to protect iso-induced myocardial injury. Previous studies (Thabassum Akhtar Iqbal et al. 2020) have demonstrated that PAE mediated the Notch1 signalling pathway to inhibit cardiac fibrosis and reverse adriamycin-induced cardiac remodelling.

Studies (Martinez et al. 2016) have reported that MAPK signalling pathway is involved in the development of the pathological process of HF. ERK1/2 and JNK, as key signalling molecules in the MAPK family, the activation could directly mediate inflammation, cardiomyocyte hypertrophy, myocardial apoptosis and other physiological processes. Activation of ERK and JNK stimulates collagen expression and promotes fibroblast proliferation and fibrosis progression further leading to cardiac hypertrophy and fibrosis (Ren et al. 2017). Moreover, the ERK/JNK signalling pathway has been reported to mediate AKT-induced fibronectin expression. Based on this information, we measured the expression of AKT, p-AKT, ERK1/2, p-ERK1/2, JNK and p-JNK in the heart and found that PAE inhibited the ERK1/2/JNK signalling pathway, suggesting that inhibition of the ERK1/2/JNK signalling pathway may be a key mechanism for PAE against fibrosis and alleviate TAC-induced HF.

## Conclusions

Our data indicated that PAE improved cardiac dysfunction, attenuated cardiomyocyte hypertrophy and reduced apoptosis and inflammation. We also made further efforts to investigate the underlying mechanisms of PAE against TAC-induced myocardial fibrosis, which could be attributed to the inhibition of ERK1/2/JNK signalling pathway. This study suggested that PAE may be a promising traditional Chinese medicine against myocardial fibrosis and apoptosis, which also provides a new strategy for the treatment of HF.

## References

[CIT0001] Asselin CY, Lam A, Cheung DYC, Eekhoudt CR, Zhu A, Mittal I, Mayba A, Solati Z, Edel A, Austria JA, et al. 2020. The cardioprotective role of flaxseed in the prevention of doxorubicin- and trastuzumab-mediated cardiotoxicity in C57bl/6 mice. J Nutr. 150(9):2353–2363. 32510147 10.1093/jn/nxaa144

[CIT0002] Bacmeister L, Schwarzl M, Warnke S, Stoffers B, Blankenberg S, Westermann D, Lindner D. 2019. Inflammation and fibrosis in murine models of heart failure. Basic Res Cardiol. 114(3):19–756. 30887214 10.1007/s00395-019-0722-5

[CIT0003] Choy KW, Murugan D, Mustafa MR. 2018. Natural products targeting ER stress pathway for the treatment of cardiovascular diseases. Pharmacol Res. 132:119–129. 29684674 10.1016/j.phrs.2018.04.013

[CIT0004] Gao L, Wang Z, Lu D, Huang J, Liu J, Hong L. 2019. Paeonol induces cytoprotective autophagy via blocking the Akt/mTOR pathway in ovarian cancer cells. Cell Death Dis. 10(8):609–621. 31406198 10.1038/s41419-019-1849-x PMC6690917

[CIT0005] Jia Q, Wang L, Zhang X, Ding Y, Li H, Yang Y, Zhang A, Li Y, Lv S, Zhang J. 2020. Prevention and treatment of chronic heart failure through traditional Chinese medicine: role of the gut microbiota. Pharmacol Res. 151:1016–1041. 10.1016/j.phrs.2019.104552 31747557

[CIT0006] Jin X, Wang J, Xia ZM, Shang CH, Chao QL, Liu YR, Fan HY, Chen DQ, Qiu F, Zhao F. 2016. Anti-inflammatory and anti-oxidative activities of paeonol and its metabolites through blocking MAPK/ERK/p38 signaling pathway. Inflammation. 39(1):434–446. 26433578 10.1007/s10753-015-0265-3

[CIT0007] Li H, Song F, Duan LR, Sheng JJ, Xie YH, Yang Q, Chen Y, Dong QQ, Zhang BL, Wang SW. 2016. Paeonol and danshensu combination attenuates apoptosis in myocardial infarcted rats by inhibiting oxidative stress: roles of Nrf2/Ho-1 and PI3K/Akt pathway. Sci Rep. 6:23693. 27021411 10.1038/srep23693 PMC4810373

[CIT0008] Lu L, Qin Y, Chen C, Guo X. 2018. Beneficial effects exerted by paeonol in the management of atherosclerosis. Oxid Med Cell Longev. 2018:1098617. 30524649 10.1155/2018/1098617 PMC6247470

[CIT0009] Martinez PF, Bonomo C, Guizoni DM, Junior SA, Damatto RL, Cezar MD, Lima AR, Pagan LU, Seiva FR, Bueno RT, et al. 2016. Modulation of MAPK and NF-954;B signaling pathways by antioxidant therapy in skeletal muscle of heart failure rats. Cell Physiol Biochem. 39(1):371–384. 27351177 10.1159/000445631

[CIT0010] Mei L, He M, Zhang C, Miao J, Wen Q, Liu X, Xu Q, Ye S, Ye P, Huang H, et al. 2019. Paeonol attenuates inflammation by targeting Hmgb1 through upregulating Mir-339-5p. Sci Rep. 9(1):19370–19384. 31852965 10.1038/s41598-019-55980-4 PMC6920373

[CIT0011] Okin PM, Devereux RB, Liu JE, Oikarinen L, Jern S, Kjeldsen SE, Julius S, Wachtell K, Nieminen MS, Dahlöf B. 2004. Regression of electrocardiographic left ventricular hypertrophy predicts regression of echocardiographic left ventricular mass: the life study. J Hum Hypertens. 18(6):403–409. 15057252 10.1038/sj.jhh.1001707

[CIT0012] Ren J, Zhang N, Liao H, Chen S, Xu L, Li J, Yang Z, Deng W, Tang Q. 2017. Caffeic acid phenethyl ester attenuates pathological cardiac hypertrophy by regulation of MEK/ERK signaling pathway *in vivo* and vitro. Life Sci. 181:53–61. 28449869 10.1016/j.lfs.2017.04.016

[CIT0013] Suematsu Y, Jing W, Nunes A, Kashyap ML, Khazaeli M, Vaziri ND, Moradi H. 2018. Lcz696 (sacubitril/valsartan), an angiotensin-receptor neprilysin inhibitor, attenuates cardiac hypertrophy, fibrosis, and vasculopathy in a rat model of chronic kidney disease. J Card Fail. 24(4):266–275. 29325796 10.1016/j.cardfail.2017.12.010

[CIT0014] Sun Y, Liu WZ, Liu T, Feng X, Yang N, Zhou HF. 2015. Signaling pathway of MAPK/ERK in cell proliferation, differentiation, migration, senescence and apoptosis. J Recept Signal Transduct Res. 35(6):600–604. 26096166 10.3109/10799893.2015.1030412

[CIT0015] Syeda MZ, Fasae MB, Yue E, Ishimwe AP, Jiang Y, Du Z, Yang B, Bai Y. 2019. Anthocyanidin attenuates myocardial ischemia induced injury via inhibition of Ros-Jnk-Bcl-2 pathway: new mechanism of anthocyanidin action. Phytother Res. 33(12):3129–3139. 31774233 10.1002/ptr.6485

[CIT0016] Thabassum Akhtar Iqbal S, Tirupathi Pichiah PB, Raja S, Arunachalam S. 2020. Paeonol reverses adriamycin induced cardiac pathological remodeling through Notch1 signaling reactivation in H9c2 cells and adult zebrafish heart. Chem Res Toxicol. 33(2):312–323. 31307187 10.1021/acs.chemrestox.9b00093

[CIT0017] Wang X, Zhu G, Yang S, Wang X, Cheng H, Wang F, Li X, Li Q. 2011. Paeonol prevents excitotoxicity in rat pheochromocytoma Pc12 cells via downregulation of ERK activation and inhibition of apoptosis. Planta Med. 77(15):1695–1701. 21509715 10.1055/s-0030-1271033

[CIT0018] Wu J, Sun C, Wang R, Li J, Zhou M, Yan M, Xue X, Wang C. 2018. Cardioprotective effect of paeonol against epirubicin-induced heart injury via regulating miR-1 and PI3K/Akt pathway. Chem Biol Interact. 286:17–25. 29505745 10.1016/j.cbi.2018.02.035

[CIT0019] Wu J, Xue X, Zhang B, Jiang W, Cao H, Wang R, Sun D, Guo R. 2016. The protective effects of paeonol against epirubicin-induced hepatotoxicity in 4t1-tumor bearing mice via inhibition of the PI3K/Akt/NF-kB pathway. Chem Biol Interact. 244:1–8. 26646421 10.1016/j.cbi.2015.11.025

[CIT0020] Xu CN, Kong LH, Ding P, Liu Y, Fan ZG, Gao EH, Yang J, Yang LF. 2020. Melatonin ameliorates pressure overload-induced cardiac hypertrophy by attenuating Atg5-dependent autophagy and activating the Akt/mTOR pathway. Biochim Biophys Acta Mol Basis Dis. 1866:165848. 32473999 10.1016/j.bbadis.2020.165848

[CIT0021] Yan X, Xun M, Wu L, Du X, Zhang F, Zheng J. 2018. DRm217 attenuates myocardial ischemia–reperfusion injury via stabilizing plasma membrane Na^+^–K^+^-ATPase, inhibiting Na^+^–K^+^-ATPase/ROS pathway and activating PI3K/Akt and ERK1/2. Toxicol Appl Pharm. 349:62–71. 10.1016/j.taap.2018.04.030 29702141

[CIT0022] Yang X, He T, Han S, Zhang X, Sun Y, Xing Y, Shang H. 2019. The role of traditional Chinese medicine in the regulation of oxidative stress in treating coronary heart disease. Oxid Med Cell Longev. 2019:3231424. 30918578 10.1155/2019/3231424 PMC6409025

[CIT0023] Yang ZZ, Tschopp O, Di-Poï N, Bruder E, Baudry A, Dümmler B, Wahli W, Hemmings BA. 2005. Dosage-dependent effects of Akt1/protein kinase Balpha (PKBalpha) and Akt3/PKBgamma on thymus, skin, and cardiovascular and nervous system development in mice. Mol Cell Biol. 25(23):10407–10418. 16287854 10.1128/MCB.25.23.10407-10418.2005 PMC1291243

[CIT0024] Yasuno S, Kuwahara K, Kinoshita H, Yamada C, Nakagawa Y, Usami S, Kuwabara Y, Ueshima K, Harada M, Nishikimi T, et al. 2013. Angiotensin II type 1a receptor signalling directly contributes to the increased arrhythmogenicity in cardiac hypertrophy. Br J Pharmacol. 170(7):1384–1395. 23937445 10.1111/bph.12328 PMC3838685

[CIT0025] Zhang L, Ma C, Gu R, Zhang M, Wang X, Yang L, Liu Y, Zhou Y, He S, Zhu D. 2018. Paeonol regulates hypoxia-induced proliferation of pulmonary artery smooth muscle cells via EKR 1/2 signalling. Eur J Pharmacol. 834:257–265. 30053410 10.1016/j.ejphar.2018.07.017

[CIT0026] Zhang Y, Sun L, Zhang Y, Liang H, Li X, Cai R, Wang L, Du W, Zhang R, Li J, et al. 2013. Overexpression of microrna-1 causes atrioventricular block in rodents. Int J Biol Sci. 9(5):455–462. 23678295 10.7150/ijbs.4630 PMC3654494

[CIT0027] Zhao P, Zhou WC, Li DL, Mo XT, Xu L, Li LC, Cui WH, Gao J. 2015. Total glucosides of Danggui Buxue Tang attenuate BLM-induced pulmonary fibrosis via regulating oxidative stress by inhibiting Nox4. Oxid Med Cell Longev. 2015:645814. 26347805 10.1155/2015/645814 PMC4548145

[CIT0028] Zhao Y, Jiang Y, Chen Y, Zhang F, Zhang X, Zhu L, Yao X. 2020. Dissection of mechanisms of Chinese medicinal formula Si-Miao-Yong-An decoction protects against cardiac hypertrophy and fibrosis in isoprenaline-induced heart failure. J Ethnopharmacol. 248:1120–1128. 10.1016/j.jep.2019.112050 31265887

